# Adaptive Design with Bayesian Informed Interim Decisions: Application To a Randomized Trial of Mechanical Circulatory Support

**DOI:** 10.1007/s43441-025-00861-4

**Published:** 2025-08-16

**Authors:** R. Mukherjee, N. Muehlemann, Y. Gao, Gregg W. Stone, C. Mehta

**Affiliations:** 1 MuSigmas Consultants, Barcelona, Spain; 2 Cytel, Cambridge, United States of America; 3 Abiomed, JnJ MedTech, Danvers, United States of America; 4 The Zena and Michael A Wiener Cardiovascular Institute, Icahn School of Medicine at Mount Sinai, New York, NY United States of America; 5 Department of Biostatistics, Harvard T. H. Chan School of Public Health, Boston, United States of America

## Abstract

**Background:**

Cardiovascular and oncology trials increasingly require large sample sizes and long follow-up periods. Several approaches have been developed to optimize sample size including sample size re-estimation based on the promising zone approach. With time-to-event endpoints, methods traditionally used to test for treatment effects are based on proportional hazards assumptions, which may not always hold. We propose an adaptive design wherein using interim data, Bayesian computation of Predictive Power (PP) guides the increase in sample size and/or the minimum follow-up duration.

**Methods:**

PROTECT IV is designed to evaluate mechanical circulatory support device vs. standard of care during high-risk percutaneous coronary intervention with the initial enrolment of 1252 patients and initial minimum follow-up of 12 months. The primary endpoint is the composite rate of all-cause death, stroke, durable left ventricular assist device implant or heart transplant, myocardial infarction or hospitalization for cardiovascular causes. The study will employ an adaptive increase in sample size and/or minimum follow-up at the Interim analysis. The adaptations utilize simulations to choose a new sample size up to 2500 and new minimal follow-up time up to 36 months that provides PP of at least 90%.

**Results:**

Via extensive simulations, we have examined the utility of the proposed design for situations like delayed treatment effect, early benefit only and in general crossing of survival curves. Separate Piece-wise Constant Hazard Models with non-influential (weakly-informative) Gamma-priors are fitted to the interim data for the two treatment arms, free from the proportional hazards assumptions, thus yielding more robust interim decision making. The Bayesian modeling facilitates sampling of future observations from the posterior predictive distributions with the predictive probability of trial success is computed via Monte-Carlo simulations. Simulation results show that the fitting Bayesian Piecewise Exponential models to the interim data along with the use of the posterior predictive distributions lead to more “specific” adaptation rules compared to the frequentist Conditional Power while the overall operating characteristics, type-I error and power, are similar.

**Conclusion:**

For clinical trials with time-to-event endpoints and where crossing of survival curves might be anticipated at the planning stage, flexible modeling along with wholesome use of patient-level data such as the calculation of predictive power as proposed here, may be more robust and efficient in making interim decisions such as sample size increase than the traditional use of the conditional power based on summary statistics and proportional hazards assumption.

**Supplementary Information:**

The online version contains supplementary material available at 10.1007/s43441-025-00861-4.

## Introduction

In trials where the primary endpoint is a time-to-event, statistical methodologies such as the log-rank test or the Cox proportional hazards (PH) model are traditionally used to estimate and test for treatment effect. These methodologies have optimal power and are appropriate when the assumption of proportional hazard assumption is met, which is not always the case [1, 2, 3, 4]. The issue of non-proportional hazards is becoming increasingly important in cardiovascular and oncology trials. As an example, Immune checkpoint inhibitors are known to have a delayed treatment effect as compared to chemotherapy, due to their mechanism of action [3,4]. The violation of PH assumption and crossing of Kaplan-Meier curves due to opposing trends in procedural and non-procedural infarctions have been also reported in cardiovascular trials [5, 6].

Interim monitoring and decision making may be challenging when faced with late separation between the survival curves. For the delayed treatment effect scenario, simulations show that interim analysis dominated by early events commonly met the futility criteria and resulted in high loss of power [7]. Delaying the interim analysis until data are representative of treatment effect may be too late for interim decision making. These issues have led some pharmaceutical companies to abandon futility monitoring [7].

In this article, we discuss the Bayesian predictive power as an alternative to the conditional power for making interim decisions in time-to-event trials, especially when non-proportionality of hazards is anticipated. As a case study we discuss the adaptive sample size re-estimation design for the PROTECT IV trial (NCT04763200) in which the decision to increase sample size and/or minimum follow-up time will be made at a single interim analysis using the predictive power. Although the final analysis could have been carried out under the Bayesian framework with the PWE model, it is planned to be carried out in the frequentist framework using a Cox PH model or logistic regression in case major violation of the PH assumption is observed in the data in line with current regulatory practice. We describe the Bayesian Predictive Power (PP) via an illustrative simulated dataset and extensive numerical study results covering scenarios with and without proportional hazards. Details for computation of the Bayesian PP and a comparison with the traditional conditional power (CP) are provided in the appendix.

## PROTECT IV Trial Design

PROTECT IV is a prospective, multicenter, randomized, parallel-controlled, open-label (with sponsors being blinded) trial designed to evaluate mechanical circulatory support with the Impella^®^ CP (or Impella^®^ 2.5) device (Abiomed Inc., Danvers, MA), vs. standard of care during percutaneous coronary intervention (PCI) in high-risk patients with complex coronary artery disease and reduced left ventricular function. Patients will be randomized to PCI with Impella^®^ or to a standard of care PCI with or without an intra-aortic balloon pump (IABP). The primary endpoint is the composite rate of all-cause death, stroke, durable left ventricular assist device (LVAD) implant or heart transplant, myocardial infarction (MI) or hospitalization for cardiovascular (CV) causes, assessed when the last patient enrolled reaches the minimum follow-up. The study plans to enroll a minimum of 1252 patients over 24 months with a minimum follow-up of 12 months and with $\:510$ expected primary endpoint events. The primary hypothesis is that the hazard-ratio (HR) for the above mentioned primary endpoint, Impella^®^ CP vs. standard of care is less than 1.

Assuming a control arm hazard of 1.2% per patient-month for the first 12 months, 1.7% per patient-month between months 12 and 24 and 2.9% per patient-month thereafter and proportional hazards for the investigational arm, the planned sample size of 1252 patients and a minimum follow-up of 12 months will provide power 90% power to detect a hazard ratio of 0.75 with a 2.5% one-sided type-I error using Cox PH regression. A recruitment period of 24 months is anticipated which means the planned minimum duration of the trial is around 36 months.

At the design stage, the following deviations from the above assumptions have been anticipated:

- The observed hazard rates over time may differ from the above assumption. A lower rate would necessitate a larger sample size or a longer follow-up;
- The hazard-ratio may vary over time (non-proportionality of hazards) which also includes the scenario of a delayed treatment effect;
- The hazard-ratio may be slightly higher than the assumed 0.75 but still clinically meaningful;
- The recruitment rate might be slower than planned. In this case it would be more practical to increase the minimum follow-up unless most events of interest occur relatively earlier in the follow-up.

With uncertainties around the above design parameters, an adaptive design with an interim unblinded sample size re-estimation which may result in an increased number of patients and/or an increased minimum follow-up time has been proposed as a risk-mitigation strategy.

### Interim Adaptations

A single interim analysis has been planned for the PROTECT IV trial when approximately 85% of the planned 1252 patients have been randomized. Note that 85% is not the information fraction here. At this interim the Bayesian PP will be computed in order to make interim decisions. The Bayesian PP can be interpreted as an predictive estimate of the chance of success at the final analysis based on interim data. Mathematically, it can be defined as the average conditional power, averaging over the current belief about the unknown parameters of interest in terms of their (posterior) probability distributions and can be expressed as a function of design parameters [8, 9]. The interim decision for this study involves adapting (increasing) two possible design parameters: the final sample size ($\:N$) and the minimum follow-up time ($\:F$) and thus we will denote $\:PP$ as a function of sample $\:N$ and $\:F$, i.e., $\:PP\equiv\:PP\left(N,F\right)$. Adaptations in $\:N\:$and $\:F$ will be carried out under the constraints: $\:N\le\:2500,\:F\le\:36$ months.

The interim analysis will start with computing the predictive power at the planned end of trial, i.e., at planned values of the $\:N$ and $\:F$, i.e., $\:P{P}_{\text{plan}}=PP\left(N=1250,F=12\right)$.

The adaptation rules for the PROTECT IV trial are presented in Table 1.

**Table 1:** Interim sample size Re-estimation rules based on predictive power

Interim Results	Adaptation Rule
*PP*_plan_ ≥ 0.9	Continue trial as planned. Note that 0.9 is the target power for trial
*PP*_plan_ < 0.5	Continue as planned
0.5 ≤ *PP*_plan_ < 0.9	Interim results fall in the "promising-zone". In this case increase sample size to N^*^ and/or the minimal follow-up time to F^*^ such that *PP*(N^*^, F^*^) is as close to 90% as possible within the constraints that N^*^ ≤ 2500 and F^*^ ≤ 36 months.

Note that a futility threshold could have been further introduced to the case when *PP*_plan_ < 0.5 however no futility stopping has been planned for this trial

## Modelling of the Event Times Data and Bayesian Predictive Power

**Fig. 1:**
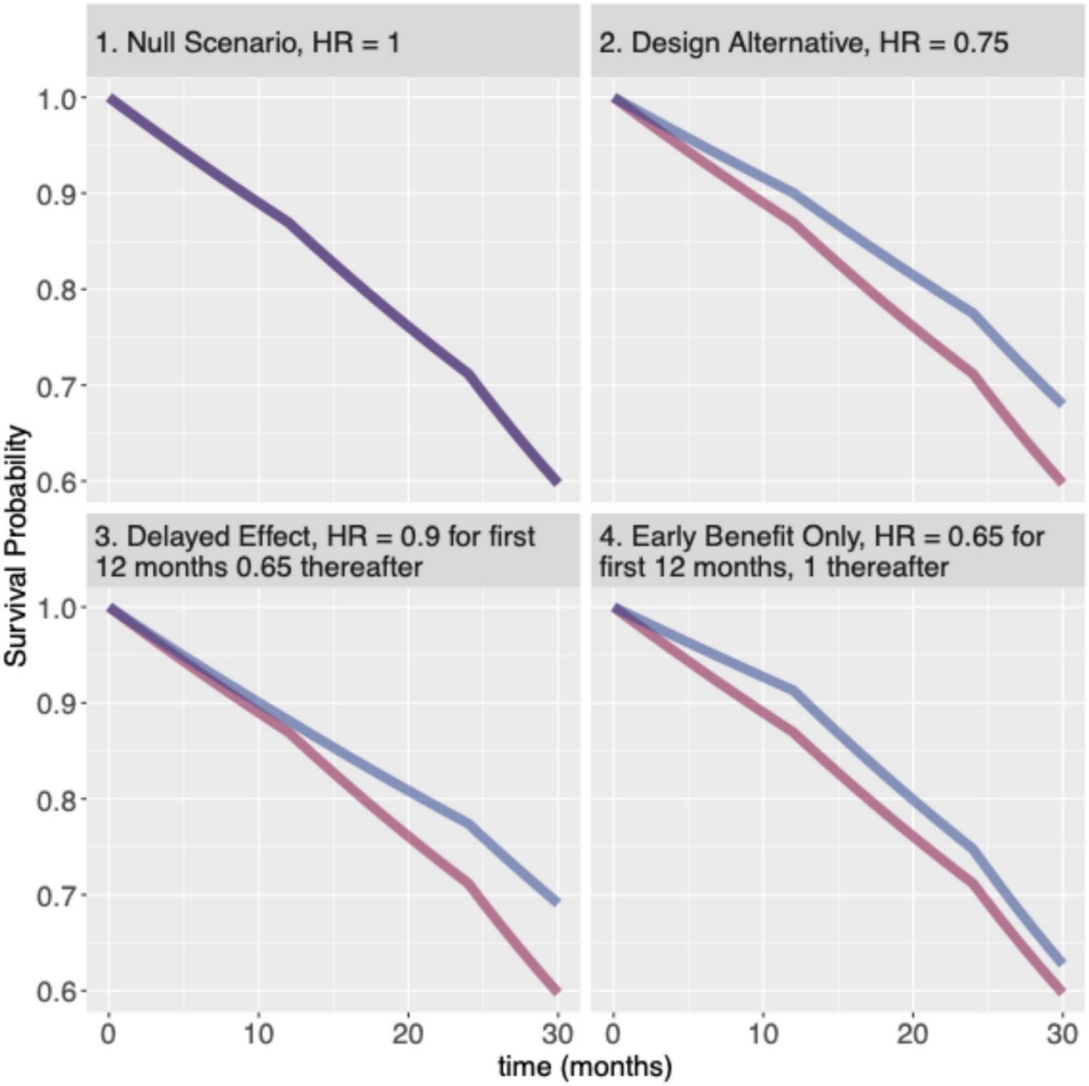
Scenarios considered for numerical study. Control arm hazard rate taken as 0.012 per month for the first 12 months, 0.017 per month for months 12 to 24 and 0.029 per month thereafter while the hazard rates for the treatment arm is given by the hazard-ratio (HR). Red line indicates control and blue line indicates treated groups

In this section we describe the modeling of the event time data and the calculation of the Bayesian PP, without technical details. We illustrate the idea using one simulated data set from the delayed treatment effect scenario. Other scenarios, including the null (hypothesis) scenario of no treatment benefit and the design alternative of 0.75 hazard-ratio (HR), considered for the overall numerical study are given in Fig. 1 below along with the theoretical survival curves from a piece-wise exponential distribution.

The Bayesian PP calculation at the interim analysis starts with assuming a piece-wise exponential (PWE) model for the event time distribution and a separate PWE model for the censoring time distribution for the interim data. Separate PWE models are assumed for the treatment and the control arms with the restriction that the two models have the same time intervals. This separation allows us to avoid any functional relationship, for example, proportional hazards, between the treatment and the control arms. The data-dependent process of choosing the number of pieces for the PWE model at the interim is pre-specified as described in Appendix A4. Note that the final analysis will not assume this model and the analysis will be carried out using the traditional baseline-covariate adjusted Cox proportional hazard model.

In the Bayesian framework, the PWE model parameters are treated as random variables unlike the frequentist framework where they would be treated as fixed but unknown. Thus, before data collection, it is customary to assume a prior distribution (based on prior belief or data) to these random variables. Once the trial data is available the data likelihood is mathematically mixed with the prior distribution using Bayes’ theorem to obtain the posterior distribution which is then used for inference and decision-making. For an introduction to Bayesian medical statistics we refer the interested reader to [10]. For the PROTECT IV interim analysis, we assume a weakly informative Gamma prior (described in the Appendix) for the hazard rates of interest.

The Gamma distribution used to specify the priors here are governed by two parameters: the rate (or the number of events) and the exposure time.

Under the PWE model with the data likelihood and the Gamma prior lead to a conjugate posterior which is also a Gamma distribution whose parameters are governed by the number of events and exposure in each of the intervals.

## Simulations To Inform Trial Design

Extensive numerical studies were carried out to establish the operating characteristics of the proposed adaptive design using the Bayesian PP to make adaptive increase in sample size and/or minimal follow-up time. Several scenarios listed in Fig. 1 are investigated in the numerical study, including the null and the design alternative scenarios under the PH assumption as well as scenarios where the PH assumption is violated. To understand the benefits of the proposed design under non-proportionality of hazards it is important to compare the operating characteristics of the proposed design to that of a traditional (frequentist) adaptive design with sample size re-estimation using CP. Description of this comparator frequentist design is provided in Appendix. Power of a fixed design, i.e. without any interim adaptation is also included in the simulation results in the section below.

First we illustrate the computation of Bayesian PP for the delayed treatment effect scenario in Fig. 1 using one simulated dataset. Readers interested in mathematical details of the PP computation can find more information in the Appendix.

### Illustrating Predictive Power Computation, Interim Decision, and Final Analysis for the Delayed Effect Scenario

Using one simulated data set under the delayed treatment effect scenario (HR is 0.9 for the first 12 months and 0.65 thereafter, scenario 3 in Fig. 1), we illustrate the computation of the Bayesian PP and how an interim decision to increase the sample size and/or the minimal follow-up times can be aided using the computed predictive powers and a grid search ranging from the planned to the maximum sample size and minimal follow-up. Note that the dataset chosen here for illustration is such that the frequentist CP described in Appendix is small (below the promising-zone) while the PP is in the promising-zone. This situation is not an unlikely one as can be seen from the scatter plot of CP vs. PP for 100 simulated data under the delayed treatment effect scenario in Figure A2 (Appendix).

#### Predictive Power

In Sect. 2.1 we introduced the Bayesian PP as an average conditional power. To be more precise the PP is the expected conditional power where the expectation is taken over the posterior distribution of the parameters of interest based on the interim data. In our case, the parameters of interest are the piecewise hazard rates for the two arms corresponding to the piecewise exponential model. In general terms, the CP is defined as the probability of showing statistical significance at the final analysis given the interim data. The CP in the frequentist framework has a closed form mathematical expression in terms of the interim log-rank z-statistic, the interim hazard-ratio estimate and the final planned number of events of interest while the Bayesian PP needs to be computed using Monte-Carlo methods.

**Fig. 2:**
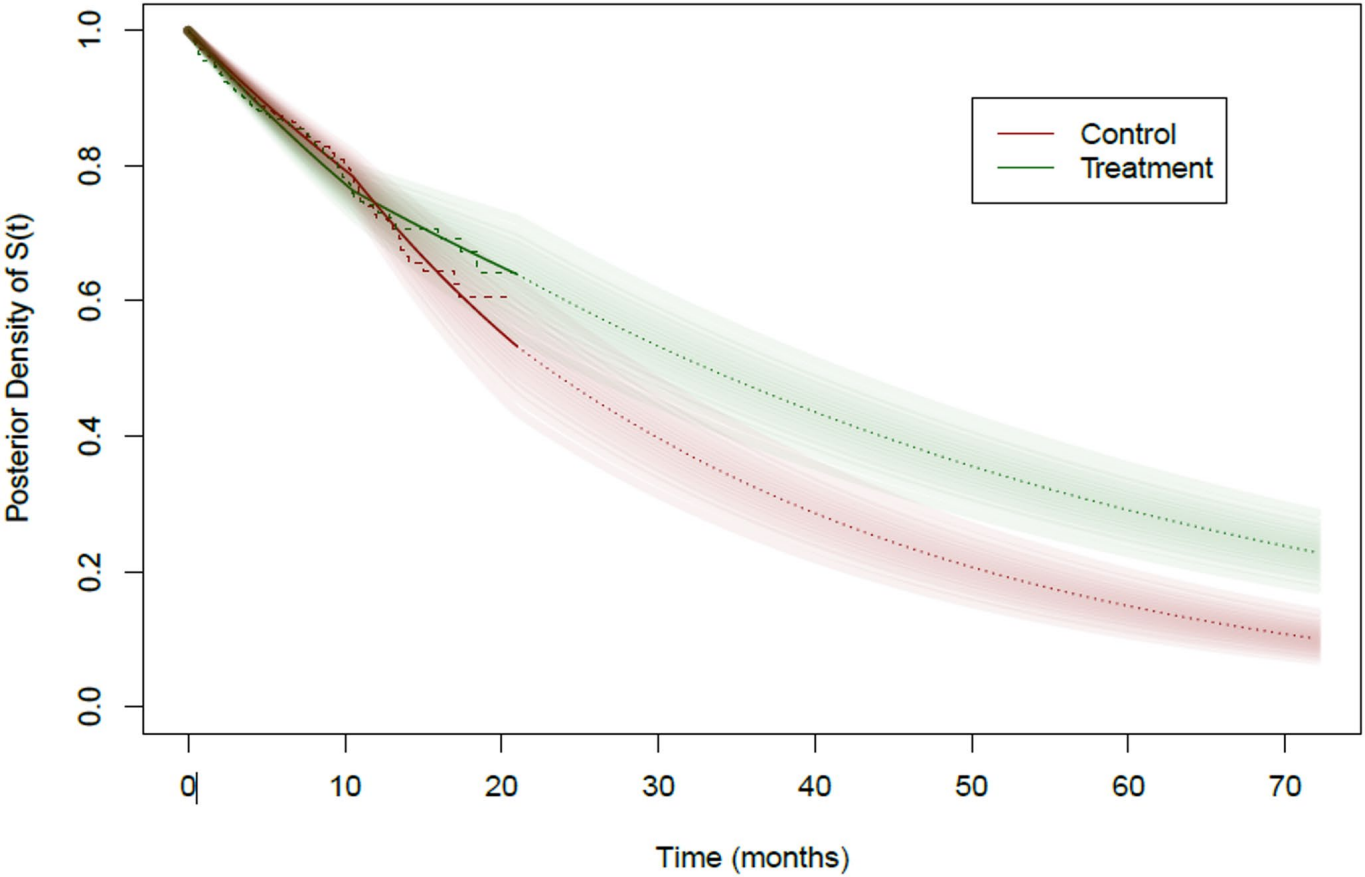
Posterior bands for Survival functions. Green: Treatment, Red: Control. Solid dark lines are posterior medians till 21 months, dotted lines are extrapolations for the median survival curve beyond 21 months using the posterior distribution of piecewise hazards while dashed lines are Kaplan-Meier plots till 21 months

For the illustrative dataset of the delayed treatment effect scenario, the interim analysis is carried out at 21 months calendar time with 1077 randomized patients (86% of the planned 1252) and with 200 primary endpoint events, 35 early withdrawals and the remaining 842 patients administratively censored with incomplete follow-up. The interim estimate for the hazard ratio using a Cox PH model is 0.96 (p-value = 0.817). The Kaplan-Meier plots obtained at the interim analysis are shown in Fig. 2 using dashed lines.

The PP computation can be understood using the survival bands in Fig. 2 obtained using the Bayesian PWE model described above. See Appendix A1 on how these survival bands are computed from the posterior distribution of the piecewise hazard rate. The essence of the calculation is to use these survival bands for predicting new events to complete the incomplete data available at the interim along with predictions for patients who are yet to be enrolled. For computing the PP, one survival function is randomly drawn for each arm, from the green and red bands in Fig. 2. Using the drawn survival curves the incomplete data is completed conditional on the treatment assignment and the observed follow-up times till the interim. For example, if a patient is administratively censored at the time of the interim with an incomplete follow-up of 6 months, then the time of their event is predicted based on the drawn survival curve between 6 months and end of study. For new patients yet to be enrolled the entire drawn survival curve starting at zero is used while the recruitment rate is estimated from the interim data. Similarly, a Bayesian PWE model is also used to model the observed censoring times till the interim and used to predict censoring times for administratively censored and new patients. Once a set of predictions is made a complete dataset of required sample size is obtained. The final analysis is carried out using this complete dataset using the frequentist log-rank test to test for treatment effect in terms of the hazard ratio and a binary indicator of trial success is noted. This step of completing the incomplete data available at the interim is repeated several ($\:R\ge\:1000$) times, each time noting the binary indicator of trial success. The proportion of trial success in $\:R$ replications provides an estimate for the Bayesian conditional power for the particular pair (treatment, control) of survival curves drawn. The drawing of the pairs of survival curves is then repeated several times ($\:B\ge\:10,000$) and the Bayesian conditional power is calculated for each pair. The average of the Bayesian conditional power over all sampled survival curve pairs then provides a Monte-Carlo estimate for the Bayesian PP.

For the same simulated dataset, the CP at the planned sample size of 1252 and a minimum follow-up of 12 months and assuming proportional hazard is only 0.02, suggesting no change of trial design, and possibly stopping for futility. On the other hand, the Bayesian PP at the planned sample size and minimum follow-up is 0.746, which is in the promising zone suggesting an increase in sample size and/or minimum follow-up in order for the predictive power to achieve the target power of 90%. These results can be explained by the survival bands and the Kaplan-Meier curves till the 21 months calendar time of the interim analysis in Fig. 2. We can clearly see the crossing of the Kaplan-Meier curves around 12 months and the late separation of the curves. However, the commonly used CP does not account for this late separation. Under proportional hazards assumption this scenario results in an interim HR estimate close to 1 suggesting no treatment effect and thus illustrating the fact that making interim decisions based on the CP in scenarios where proportionality of hazards may be violated can be potentially misleading.

#### Interim Decisions Using the Predictive Power

**Fig. 3:**
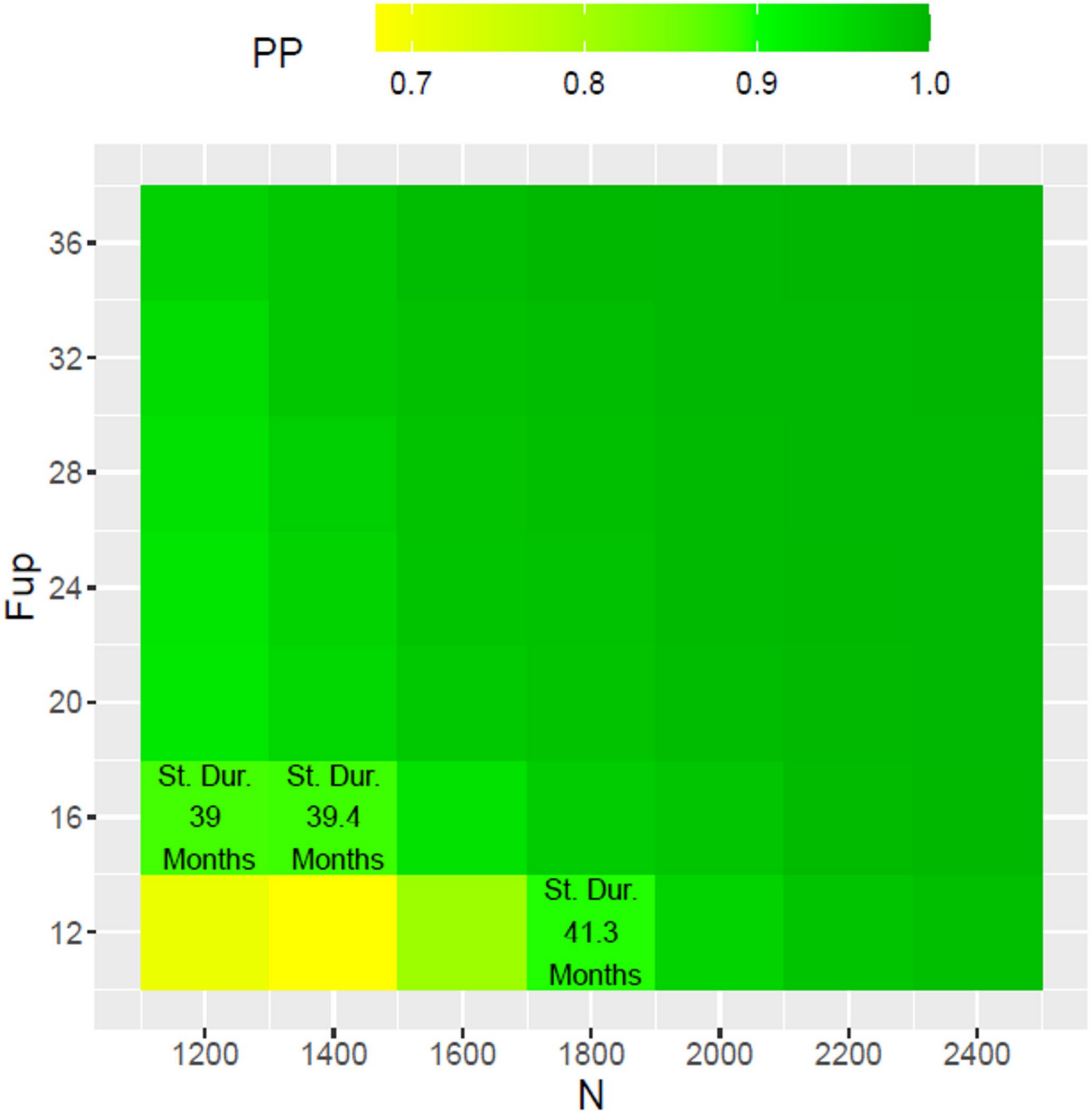
Grid Search optimizing for study duration with PP ≈ 0.9

Continuing with the simulated dataset used for illustrations in the sections above, we illustrate how the interim decision regarding the increase of sample-size and/or minimal follow-up times are made. Recall that the PP based on the planned sample-size of 1252 and a minimum follow-up of 12 months for this interim dataset is 0.746. We carry out a grid search over different sample sizes ranging from 1252 to the maximum pre-specified sample size of 2500 and different minimum follow-up ranging from 12 months to the maximum pre-specified follow-up of 36 months and calculate the PP for each combination of sample-size and minimum follow-up time. PP at each combination of sample size and minimal follow-up is shown in the heatmap in Fig. 3. The combination that yields a predictive power of at least 90% with a minimum predicted study duration, defined as enrollment time plus minimum follow up (12 months), is chosen as the optimal adaptation. Figure 3 below shows the grid search results over a coarse $\:7\times\:7$ grid. We see that the combinations hitting the 90% PP mark are $\:\left(N=1252,F=16\right)$ with a predicted study duration of 39 months, $\:\left(N=1400,F=16\right)$ with a predicted study duration of 39.4 months and $\:\left(N=1800,F=11\right)$ with a predicted study duration of 41.3 months. Note that cells above and on the right of these cells all have predictive power greater than 90%. We choose $\:\left(N=1252,F=16\right)\:$as the starting combination and via a finer grid search we get a PP of 0.919 with $\:\left(N=1400,F=15\right)$ with a predicted study duration of 38.43 months and 602 events. This is combination that minimizes the total study duration and hence used as the interim adaptation decision. With this adaptation the final analysis yields a HR estimate of 0.782 (p-value = 0.002).

### Simulations for Establishing Operating Characteristics

Here we describe the results of running several simulations under each of the scenarios in Fig. 1 in order to establish and compare the operating characteristics of the proposed adaptive design with those of a traditional adaptive design using the CP. Note that unlike the frequentist CP setting [12], there is no analytical formula for determining the PP cut-off that guarantees the preservation of the type-1 under sample size adaptation, and thus needs to be determined using extensive simulations. Our background simulations, not covered here, show that a cut-off of PP = 0.5 provides type-I error rate control.

For all the scenarios listed in Fig. 1 replications were carried out except for the null scenario for which the results are based on 10,000 replications.

The simulation results presented in Table 2 demonstrate that using the Bayesian PP approach leads to more “specific” adaptation in sample size and/or minimum follow-up compared to the frequentist CP methods. By “specific” we mean that the sample size increase is not triggered unnecessarily when the interim data exhibits evidence of non-proportionality in hazards. This is achieved via the use of flexible modeling like the use of two separate PWE models for the treatment and the control arms and also the wholesome use of patient-level data via the use of the posterior predictive distributions under the Bayesian framework. The overall operating characteristics, such as type-I error rates and statistical power under the different scenarios, remain similar between the two methods. Additionally, we observed that while the probability of interim results falling within the promising zone (as shown in Table 2. column 7) is smaller for the Bayesian PP method, there are greater gains in statistical power when sample size and/or minimum follow-up are adapted as opposed to when they are not adapted. This difference can be seen in the powers with and without adaptation when interim results fall within the promising zone (as indicated in Table 2, columns 8 and 12). In essence, this means that the adaptation process is more specific, as it is triggered only when it is most needed. There is one exception to this trend, which occurs in the early-benefit only scenario. In this case, the probability of falling within the promising zone is higher for the Bayesian PP method compared to the frequentist CP method. However, this can be explained by the fact that, under this scenario, the use of the proportional hazard assumption results in most instances where the frequentist CP is either above or below the promising zone, leading to fewer adaptation when compared to scenarios with no adaptations. This once again highlights the limitation of making interim decision based on the proportional hazards assumption, especially when it may not hold true.

**Table 2:**
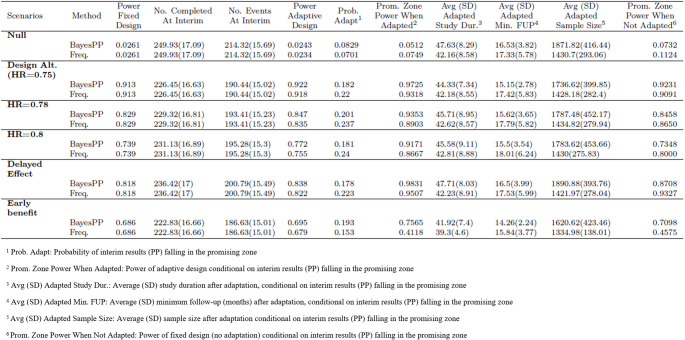
Operating characteristics of the proposed adaptive design with bayesian PP compared with standard frequentist design with adaptation based on CP

## Discussion

Adaptive sample-size re-estimation designs have been used to mitigate the risks of under powering a trial based on assumptions that may not be reflected in the observed data [11]. The promising-zone design introduced by Mehta and Pocock [12] popularized the use of the “promising-zone” when the CP at the interim analysis based on the actual planned sample size falls between 50% and target power. In that range the sample size can be increased up to a maximum pre-specified without the need for special, non-standard data analysis. In this article, we discuss the use of the Bayesian PP as an alternative to the CP, however, working within the premise of the promising-zone approach. Using extensive simulations we are able to identify and discuss the potential pitfalls of carrying out sample-size re-estimation based on the CP for time-to-event trials where proportional hazards or time-constant hazards assumptions may be violated. We propose the use of the piece-wise exponential model that may be used to counter the fact that hazards may not be constant over time. Using separate PWE models for the treatment and control arms we are able to avoid the need for making a relational assumption between the hazards in the two arms. The pre-specification for PWE models requires the use of same time intervals for the two arms. This requirement provides computational simplicity as well as greater insight into the interim data. For example, in order to extrapolate the data to follow-up time beyond the interim calendar time and to be able to detect trends like delayed treatment effect in terms of piece-wise posterior distribution of the hazard ratio. Although the final analysis for the Protect IV trial has been planned as a frequentist log-rank test, if we were to conduct the analysis in the Bayesian framework, this constraint of “same time intervals” would be needed, for example, for testing treatment effects using the posterior probability that the maximum hazard ratio (over all intervals) is less than one.

As illustrated here, the Bayesian predictive power calculation can take into account the fact that the final analysis will be based on a Cox PH model or a log-rank test as planned for the PROTECT IV trial and in line with current regulatory practices. Indeed, a fully Bayesian final analysis would have been a more consistent and preferred approach, however, the hybrid-approach provides a reasonable compromise between the ability to make flexible and informed interim decisions while adhering to current regulatory practices for registrational trials.

Using a clinically realistic scenario with late separation of survival curves, we illustrated how interim results based on the CP under the PH assumption can in some cases suggest stopping for futility even though the final results may end up to be statistically significant. For such scenarios interim decision-making based on pre-specified CP becomes difficult, especially since the late separation may be clearly evident from the interim data. This depends, however, on the timing of the interim analysis relative to the delay in separation of the survival curves. The use of PP as an alternative to CP can be planned for if such trends have been observed in previous trials. The operating characteristics of such designs will need to be investigated using extensive simulations as was done for the study design of the PROTECT-IV trial discussed here.

The possible adaptation for the PROTECT-IV trial includes an increase in sample-size and/or an increase in minimum follow-up time. This is motivated by the fact that hazard rates for the two arms may not be constant over time. For example, if the interim data suggest that HR is low (i.e. greater early treatment effect than anticipated) at the beginning then enrolling additional subjects rather than increasing the minimum follow-up time may lead to a shorter study duration. Conversely, prolonging follow-up times may be less costly and time-saving compared with enrolling more patients if the observed HR is low late in the follow-up period. This also necessitates that the interim analysis be carried out as late as possible.

This article provides an example of how informed interim decisions can be made via the application of Bayesian methodology and use of flexible models to overcome pitfalls of traditional methods when non-proportionality of hazards are anticipated. The sensitivity of the proposed predictive power approach to recognize deviations from proportionality in hazards comes from the use of the flexible PWE models. Indeed, other flexible models could also be used. The Bayesian computations on the other hand, use patient-level data and provide means to predict future events and censoring times as well as to incorporate the uncertainty in estimation of the survival curves at the interim look.

## Supplementary Information

Below is the link to the electronic supplementary material.

Supplementary Material 1

## Data Availability

No datasets were generated or analysed during the current study.
