# Supplementary material for: Adaptive Design with Bayesian Informed Interim Decisions: Application To a Randomized Trial of Mechanical Circulatory Support
Source: Ther Innov Regul Sci. 2025 Aug 16;59(6):1516–25. doi: 10.1007/s43441-025-00861-4 (PMC12579667; doi:10.1007/s43441-025-00861-4)
Supplement: Supplementary file 1 — Supplementary Material 1 [file 43441_2025_861_MOESM1_ESM.docx]

# Appendix

**A1. Modeling of the event time data**

The Bayesian predictive power calculation at the interim analysis starts with assuming a piece-wise exponential (PWE) model for the event time distribution and a separate PWE model for the censoring time distribution for the interim data. Suppose that the observed maximum follow-up time in the interim dataset is $\tau_{interim}$. First the time interval $(0,\tau_{\text{interim}}]$ is divided into $K$ equal-length intervals $(\tau_{k-1},\tau_{k}];k=1,2,\ldots,K$ with $\tau_{0}=0$ and $\tau_{K}=\tau_{\text{interim}}$. The PWE model for the $i^{th}$ arm ($i=0$ for control and $i=1$ for treatment) can be expressed in terms of the hazard function $h\left( t \right)$ at patient follow-up time $t$ as

$h^{i}\left( t \right)=\sum_{k=1}^{K} \lambda_{k}^{i}I_{(\tau_{k-1},\tau_{k}]}\left( t \right),$ (1)

where $\lambda_{k}^{i}$ is the constant hazard rate in the $k^{th}$ intervals for the $i^{th}$ arm. Separate PWE models are assumed for the treatment and the control arms with the restriction that the two models have the same time intervals. This separation allows us to avoid any functional relationship, for example, proportional hazards, between the treatment and the control arms. In particular, note that the time intervals given by $(\tau_{k-1},\tau_{k}]$ where we assume a constant hazard are the same for the two treatment arms, however the constant hazard rates $\lambda_{k}^{i}$ are treatment specific. The number of intervals $K$ will be determined upon inspection of the data available at the time of the interim analysis. Other choices for the selection of number of pieces in the PWE model are described in section A4. The PWE model is also assumed for time to early withdrawal (censoring) with perhaps a different $K$. In case the number of early withdrawals at the time of the interim analysis is small, an exponential distribution ($K=1$) can be assumed. Note that the final analysis will not assume this model and the analysis will be carried out using the traditional baseline-covariates adjusted Cox proportional hazard model and the log-rank test.

In the Bayesian framework, the model parameters, which for the assumed PWE model are $\lambda_{k}^{i};i=0,1,k=1,2,\ldots,K,$ are treated as random variables unlike the frequentist framework where they would be treated as fixed but unknown. For the PROTECT IV interim Bayesian analysis, we assume a weakly informative Gamma prior given by

$$\lambda_{k}^{i}\sim\text{ Gamma}\left( a=0.01,b=0.01 \right),\text{ for each }i,k.$$

Note that the prior distribution choice is not unique, however the assumed Gamma prior is a weakly informative prior in the sense that it will very rapidly be dominated by the target trial interim data. The Gamma distribution used to specify the priors here are governed by two parameters: the rate and the exposure time.

Under the PWE model with piece-wise exponential data likelihoods and the Gamma prior leads to a conjugate posterior which is also a Gamma distribution whose parameters are governed by the rates and exposure in each of the intervals.

Note that the hazard rates $\lambda_{k}^{i}$ over the $K$ intervals only span the maximum observed follow-up time ($\tau_{K}$) till the time of the interim analysis. Thus, assumptions need to be made on the distribution of hazard rates for the unobserved future interval from $\tau_{K}$ to the duration of the trial ($\tau$), i.e., for the time interval $(\tau_{K},\tau]$. We propose setting the distribution of the future hazard rates for the two arms to a Gamma distribution with rates equal to the weighted average of the posterior means of the hazard rates in each interval with the weights being proportional to the order of the intervals. For example, if the number of intervals selected is $K=3$ then the weights are respectively 1/6, 2/6 and 3/6 for the first, second and third intervals. Thus, the last interval leading up to the time of the interim analysis gets the maximum weight. Other possibilities have been explored including the choice of the mean hazard rate in the last observed interval, however the above choice remained the most robust across varying assumptions considered for simulations of the trial design. The exposure parameter for the Gamma posteriors are estimated based on the survival estimates at time $\tau_{K}$, the hazard rates assumed for $(\tau_{K},\tau]$ as described above and the number of patients that have incomplete data at the time of the interim and the number of future patients yet to be enrolled. More details on how these future projections to $(\tau_{K},\tau]$ are made based on available data at the time of the interim analysis are described below. Note that the process of obtaining the posteriors and projecting to the future are carried out separately for the two arms, although the time intervals remain the same.

## A2. Posterior Projection to the Future

The posterior distribution of the piece-wise hazard rates is obtained till the maximum observed follow-up time at the interim ($\tau_{K}$). Using the mathematical relationship between hazard ($h\left( t \right)$) and survival functions ($S\left( t \right)$) given by

$$S\left( t \right)=exp\left\{ -\int_{0}^{t} h\left( u \right)du \right\},$$

and using the PWE model in (1), the Gamma posteriors for the hazard rates $\lambda_{k}^{i}$ shown in Figure A1 can be combined to obtain posterior distribution bands for the survival functions for each arm as shown in Figure [2](#PostSurv) along with the observed Kaplan-Meier plots till 21 months which is the time of the interim analysis. For example, the solid lines in Figure [2](#PostSurv) representing the median of the survival bands can be obtained using the median hazard rates for the two arms from the two time-intervals till 21 months and combining the PWE model in (1) along with above relationship between $h\left( t \right)$ and $S\left( t \right)$.

These posterior survival bands obtained at the interim are used to make the projection to the future based on the currently enrolled $N_{a}$ patients who are administratively censored at the time of the interim look. Here we describe the procedure to estimate the rate and the exposure parameters for the Gamma posterior projection to the future, i.e., in the interval $(\tau_{K},\tau]$ where $\tau$ is the total duration of the study. For the sake of brevity, we describe the procedure while dropping the treatment indicator subscript ($i$). The rate parameter ($\lambda_{(\tau_{K},\tau]}^{*}$) is taken as the weighted mean of the posteriors means of the hazard pieces obtained till $\tau_{K}$ with the last interval $(\tau_{K-1},\tau_{K}]$ getting the maximum weight, specifically the rate parameter is calculated as

$$\lambda_{(\tau_{K},\tau]}^{*}=\frac{\sum_{k=1}^{K} k\hat{\lambda}_{k}}{\sum_{k=1}^{K} k}.$$

The exposure parameter (E^*^_(_$\tau$_K,_ $\tau$_]_) is estimated using the interim estimates obtained till time $\tau_{K}$. Suppose that the $N_{a}$ patients have been administratively censored at times $t_{1},t_{2},\ldots,t_{N_{a}}$ at the time of the interim. The probability of a patient with observed incomplete follow-up of $t_{j}$ to survive till time $\tau_{K}$ is $S\left( \tau_{K} \right)/S\left( t_{j} \right)$, where $S$ is the survival function. Thus, the total number of patients out of the $N_{a}$ patients expected to survive at time $\tau_{K}$ is given by

$$N_{\tau_{K}}^{*}=\sum_{j=1}^{N_{a}} \frac{S\left( \tau_{K} \right)}{S\left( t_{j} \right)},$$

where $S\left( t \right)$ is calculated using the PWE model given by

$$S\left( t \right)=exp\left\{ -\int_{0}^{t} \sum_{k=1}^{K} \lambda_{k}I_{\left( \tau_{k-1},\tau_{k} \right)}\left( u \right)du \right\}.$$

Some of these $N_{\tau_{K}}^{*}$ patients will experience the event of interest in the interval $(\tau_{K},\tau]$ with probability $1-S\left( \tau\right)/S\left( \tau_{K} \right)$. If we assume that the event times are uniformly distributed in the interval $(\tau_{K},\tau]$ then the expected exposure for these patients who experience the event in $(\tau_{K},\tau]$ can be approximated by $\left( \tau-\tau_{K} \right)/2$. Patients who survive till time $\tau$ get the full exposure of length $\tau-\tau_{K}$. Thus, the expected exposure in the future interval $(\tau_{K},\tau]$ can be approximated by

$$E_{(\tau_{K},\tau]}^{*}=\frac{1}{2}N_{\tau_{K}}^{*}\left( \tau-\tau_{K} \right)\left[ 1+\frac{S\left( \tau\right)}{S\left( \tau_{K} \right)} \right].$$

Following the PWE model and the assumed rate of $\lambda_{(\tau_{K},\tau]}^{*}$ for the future, the exposure can be easily computed as

$$E_{(\tau_{K},\tau]}^{*}=\frac{1}{2}N_{\tau_{K}}^{*}\left( \tau-\tau_{K} \right)\left\{ 1+exp\left[ -\lambda_{(\tau_{K},\tau]}^{*}\left( \tau-\tau_{K} \right) \right] \right\}.$$

## Using the simulated dataset under the delayed treatment effect (described in the section Illustrating Predictive Power computation, Interim Decision and Final Analysis for the delayed effect scenario) the posterior projection to the future in terms of the Gamma distributions are shown in Figure [A1](#PostLambdas) below where the dashed lines depict the posterior projection with $\boldsymbol{\tau}\mathbf{=72}$. The choice of $\boldsymbol{\tau}$ is somewhat arbitrary, the 72 months being based on a recruitment period of 36 months plus a maximum of 36 months of minimum follow-up time.

*Figure A1.* Posterior distribution of piece-wise ($K=2$ pieces) hazard rates under the PWE model for the two treatment arms


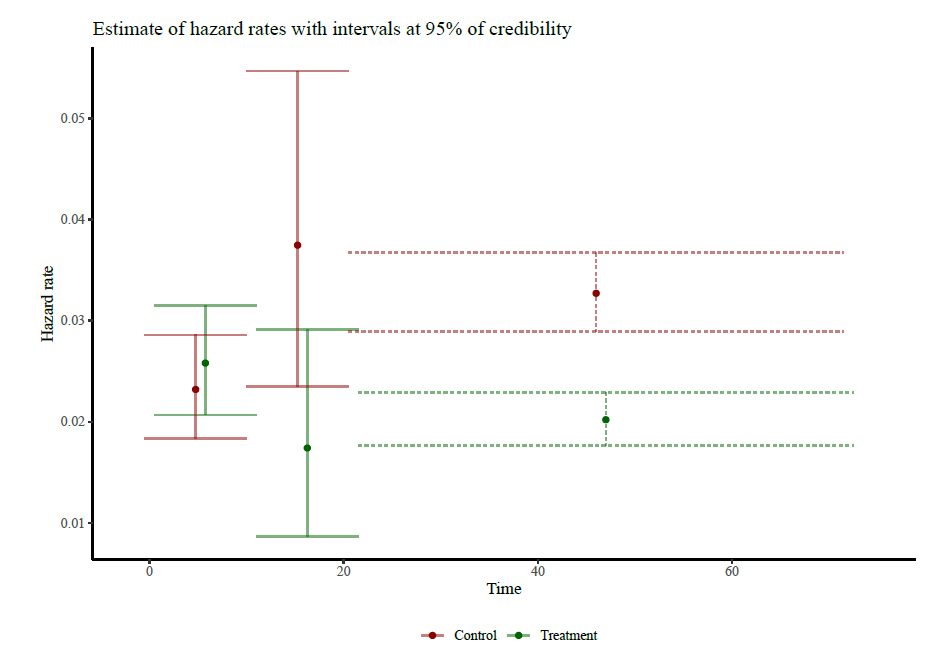


Red: control, green: treatment. Sots show the posterior mean along with the 95% credible intervals. The dotted intervals show the "projected-posterior" for the future (time beyond the interim analysis calendar time).

## A3. Simulation Results: Comparison of Conditional and Predictive Powers

For the sake of comparing the operating characteristics of our proposed Bayesian design to a more traditional frequentist adaptive design we choose an adaptive SSR design with some minor modifications. Under the frequentist adaptive design, the decision to increase the sample size is based on the Conditional Power at the planned final number of events ($\left( E_{\text{plan}} \right)$ , given the interim data.

The conditional power at the planned number of events ($CP\left( E_{\text{plan}} \right)$) is calculated using the number of events observed at the interim ($E_{I}$), the hazard ratio estimate at the interim ($\hat{\theta}_{I}$) and the log-rank statistic calculated at the interim ($Z_{I}$) with the following asymptotic formula

$$CP\left( E_{\text{plan}} \right)=\Phi\left( \frac{Z_{I}\sqrt{E_{I}}-z_{1-\alpha/2}\sqrt{E_{\text{plan}}}+\hat{\theta}_{I}\left( E_{\text{plan}}-E_{I} \right)}{\sqrt{E_{\text{plan}}-E_{I}}} \right),$$

where $z_{1-\alpha/2}$ is the $\left( 1-\alpha/2 \right)\times100\%$ quantile under the standard normal distribution and $\Phi$ is the cumulative distribution function for the standard normal distribution. If $0.5\leq CP\left( E_{\text{plan}} \right)<0.9$ then the required number of events was increased to $E_{\text{new}}$ such that $CP\left( E_{\text{new}} \right) \geq0.9$. Note that in frequentist design with time-to-event endpoints and SSR at the interim, typically a constant hazard rate is assumed to translate $E_{\text{new}}$ to the new increased sample size, for example using the pooled hazard rate. In our case, since we are interested in potentially increasing both the sample size and the minimal follow-up time we rely on the Bayesian model and prediction as described in section [3](#BPP) to obtain the optimal combination $\left( N_{new},F_{new} \right)$ that achieves $E_{\text{new}}$ events while having the smallest study duration.

Simulation results in terms of design operating characteristics shown in Table 2 are summarized over 1,000 replications (10,000 for the null scenario). In order to compare conditional and predictive powers computed at the interim, we consider scatter plots of the two for the first 100 replications for two proportional hazard scenarios (Null and design alternative of HR = 0.75) along with two scenarios under non proportionality of hazards (delayed treatment effect and early benefit only). These scenarios are described in Figure 1. Figure A2 below shows the scatter plots of CP vs. PP for the four different scenarios. For the scenarios under proportional hazards (top row), the points are more or less evenly distributed around the ${45}^{\circ}$ line with a slight propensity of the PP points to be above the ${45}^{\circ}$ line for the Null scenario. This can also be seen for the Null scenario in Table 2, where the probability of adaptation is around 8.3% using the PP while it is around 7% using the CP.

For the non-proportional hazard scenario with delayed treatment effect, we see that the PP is more optimistic than the CP while for the early benefit only the PP is less optimistic than the CP suggesting that the PP is better equipped in identifying a late separation of the survival curves or, as in the latter case, survival curves coming together after an early separation. This sensitivity of the PP to recognize deviations from proportionality in hazards comes from the use of the flexible PWE models. The Bayesian computations on the other hand, use patient-level data and provide means to predict future events and censoring times as well as to incorporate the uncertainty in estimation of the survival curves at the interim look.

*Figure A2.* Scatter plots of CP (x-axis) vs. PP (y-axis) for different scenarios under proportional and non-proportional hazards
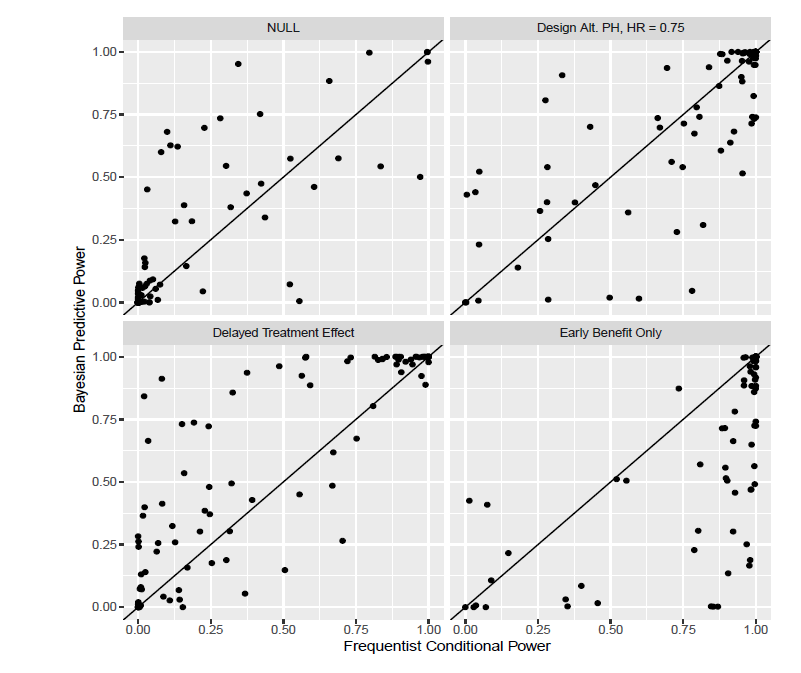


##

## A4. Selecting the Number of Pieces ($\boldsymbol{K}$) for the PWE model

As mentioned in Section A[3](#BPP), the number of pieces for the PWE model fitted to the events and censoring for early withdrawal data available at the time of interim is determined upon inspection of the data and the process is pre-specified in the trial protocol and the statistical analysis plan. Specifically, the time interval $(0,\tau_{0}]$, with $\tau_{0}$ being the maximum observed follow-up time available within the interim data is divided into $K$ intervals of equal lengths. $K$ is chosen such that there are at least 10% of all observed events in each interval in each treatment arm. Thresholds higher than the 10% used here have also been explored and simulation results (not presented here) show that the predictive power computation is fairly robust against the particular threshold value as long as it is around the 10% mark. Note that the restriction of using the same number of pieces ($K$) for the two treatment arms is not necessary and has only been considered for the ease of comparability of the two arms in an unblinded fashion. Other options including using a full Bayesian hierarchical model with a prior on $K$ or using optimal (in terms of coverage probabilities) values between $K=\left( n/logn \right)^{1/2}$ and $K=\left( n/logn \right)^{1/3}$ , where $n$ denotes the number of events, can also be used, however, we resort to a simple and pragmatic way of selecting the choice of the number of pieces for the purpose of the interim decision making.
